# Breakthroughs in the Design of Novel Carbon-Based Metal Oxides Nanocomposites for VOCs Gas Sensing

**DOI:** 10.3390/nano10081485

**Published:** 2020-07-29

**Authors:** Eleonora Pargoletti, Giuseppe Cappelletti

**Affiliations:** 1 Dipartimento di Chimica, Università degli Studi di Milano, Via Golgi 19, 20133 Milan, Italy; 2 Consorzio Interuniversitario per la Scienza e Tecnologia dei Materiali (INSTM), Via Giusti 9, 50121 Firenze, Italy

**Keywords:** graphene, graphene oxide, reduced graphene oxide, single-wall carbon nanotubes, multi-wall carbon nanotubes, metal oxides, gas sensing, volatile organic compounds, chemoresistor, sensing mechanism

## Abstract

Nowadays, the detection of volatile organic compounds (VOCs) at trace levels (down to ppb) is feasible by exploiting ultra-sensitive and highly selective chemoresistors, especially in the field of medical diagnosis. By coupling metal oxide semiconductors (MOS e.g., SnO_2_, ZnO, WO_3_, CuO, TiO_2_ and Fe_2_O_3_) with innovative carbon-based materials (graphene, graphene oxide, reduced graphene oxide, single-wall and multi-wall carbon nanotubes), outstanding performances in terms of sensitivity, selectivity, limits of detection, response and recovery times towards specific gaseous targets (such as ethanol, acetone, formaldehyde and aromatic compounds) can be easily achieved. Notably, carbonaceous species, highly interconnected to MOS nanoparticles, enhance the sensor responses by (i) increasing the surface area and the pore content, (ii*)* favoring the electron migration, the transfer efficiency (spillover effect) and gas diffusion rate, (iii) promoting the active sites concomitantly limiting the nanopowders agglomeration; and (iv) forming nano-heterojunctions. Herein, the aim of the present review is to highlight the above-mentioned hybrid features in order to engineer novel flexible, miniaturized and low working temperature sensors, able to detect specific VOC biomarkers of a human’s disease.

## 1. Introduction

Wearable electronics is expected to be one of the most active research areas in the near future. Hence, materials possessing elevated electronic, optical, and mechanical features alongside desired flexibility and lightweight are required [1,2]. Thus, the design of novel networks that fulfill all these requirements is still in demand for many scientific applications, extending from the environmental field [3,4,5] to food quality monitoring [6] and medical diagnosis [7,8]. In particular, concerning the latter, recent successes in non-invasive medical diagnostics, based on human breath analysis, have been pushing forward the development of extremely sensitive gas sensors for ppb detection of specific analytes (e.g., ethanol, acetone and aromatic compounds) in a complex gas mixture [9,10,11]. Indeed, the main components of breath are N_2_, O_2_, CO_2_, H_2_O, and inert gases. The remaining small fraction consists of more than 1000 different volatile organic compounds (VOCs) at parts per million (ppm) to parts per trillion (ppt) [12,13]. Compounds with relatively high concentrations in exhaled breath include ammonia (median concentration: 833 parts-per-billion, ppb), acetone (477 ppb), ethanol (112 ppb), propanol (18 ppb) and acetaldehyde (22 ppb) [14]. Alongside, it is well known that many diseases are accompanied by characteristic volatile organic compounds (VOCs) emission. Hence, their recognition can provide diagnostic clues, guide the laboratory evaluation, and affect the choice of immediate therapy. A correlation between the concentration patterns of VOCs and the occurrence of certain diseases has already been thoroughly evaluated and reviewed, as reported in Table 1.

In this context, the development of novel solid-state sensors for the selective detection of ultra-low concentrations of specific gas molecules has become increasingly mandatory and more feasible due to the recent progress in nanofabrication approaches, which are capable of providing atomic-level control of the surface composition in high surface-area/porosity detectors [24]. Common VOC sensing mechanisms for wearable and miniaturized sensors include chemoresistive semiconductors, surface acoustic wave resonators and capacitive polymers [25,26,27]. Concerning the former, the sensing mechanism is based on the conductivity change of a nanostructured sensor film as a function of the concentration of reducing/oxidizing gases present. Specifically, chemoresistors are mainly based on the adoption of metal oxide semiconductor (MOS) materials, among which SnO_2_ [28,29,30], ZnO [31,32], WO_3_ [9,33] and TiO_2_ [34,35] have been widely exploited. Nevertheless, although they are sensitive to many gases and VOCs, to date, they have not reached the required low concentration sensitivities for an application such as breath analysis. Moreover, the poor selectivity [36,37], scarce life-time and usually high operating temperatures [25,38] represent the major shortcomings of this type of detection. Thus, aimed at overcoming the above-mentioned drawbacks, the co-synthesis of metal oxides with other oxides/non-metal elements [39,40], their doping with metal nanoparticles (such as Pt or Ag) [24] and/or their coupling with carbon-based materials [26,41] can be valid alternatives.

In recent years, graphene and its derivatives, such as like pristine graphene (PG), graphene oxide (GO) and reduced graphene oxide (RGO), have been reported to enhance sensing properties [26,42,43]. High mechanical strength, good thermal stability, ballistic conductivity, high carrier mobility at room temperature, low electrical noise because of its unique 2D honeycomb lattice, as well as the large surface area [25,26] represent the unique features of these materials. In addition, the electrical properties of carbon nanotubes (CNTs) have been proved to be altered by the adsorption of gaseous species on their surface, subsequently exhibiting a p-type behavior [44]. However, gas sensors using only bare CNTs or graphene-based compounds have some limitations connected to insensitivity to certain analytes with a low adsorption energy or affinity, lack of selectivity and quite long recovery time [44]. Therefore, their surface functionalization can be an effective strategy to enhance their gas sensing features.

**Scheme 1:**
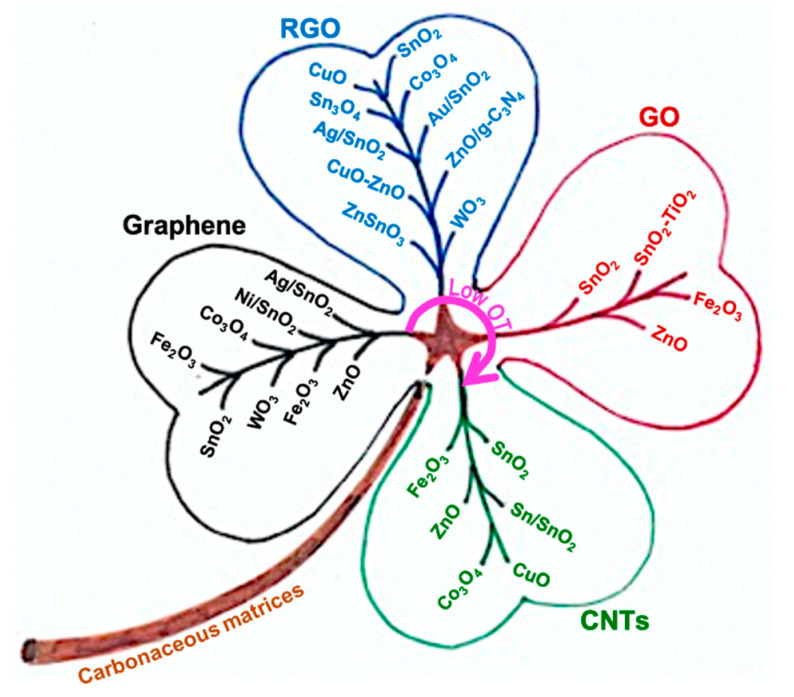
Overview of the principal composites (carbon matrices/MOS) used as volatile organic compound (VOC) sensors nowadays. OT = sensing operating temperature.

There are already several reviews aimed at highlighting the recent advancements in gas sensors based on functional materials, such as conducting polymers [45], 2D compounds [1], metal oxides [46] and so on. Nevertheless, to catalyze the investigations in this multidisciplinary area, evidencing the still opened topics to be unveiled and improved, we have attempted to show, in an organized and comprehensive way, the main breakthroughs in the engineering of carbon-based nanocomposites and their relative boosted sensing features. In particular, the present work will provide information about novel nanoarchitectures based on the exploitation of pristine graphene, graphene oxide, reduced graphene oxide or carbon nanotubes (both single-wall and multi-wall ones) coupled with at least one metal oxide semiconductor for the sensing of several VOCs. Scheme 1 provides a detailed summary of the recently used sensing materials, classified in terms of different carbonaceous matrices that will be finely reviewed in the whole text.

Along with the noteworthy experimental outcomes in terms of enhanced sensitivity, selectivity and room temperature operating conditions, mechanisms underneath the sensing behavior will be thoroughly described for each class of carbon-based supports. Finally, the remaining challenging issues to develop next-generation flexible sensors for wearable technology will be also touched on.

## 2. Chemoresistors Based on Pristine Graphene-Metal Oxides

Over the past few decades, the research interest in graphene materials has drastically risen and a plethora of studies about its use in several applications have been reported so far [6,47,48]. Additionally, in the gas sensing field, pristine graphene (G) has been considered as a promising material due to its unique electronic properties (high conductivity and ballistic transport that ensure optimal signal-to-noise ratio and do not require auxiliary electric heating thanks to its excellent chemical stability at room temperature), low cost, high surface-to-volume ratio and ease of processing [25,26,49]. The first graphene-based gas sensor was reported in 2007 by Novoselov’s group [50], who demonstrated that the adsorption of gas molecules changes the graphene local carriers concentration, thus resulting in a resistance variation. These electrical changes had different magnitudes for different gases and the sign of the change indicated whether the gas was an electron acceptor (resistance increase e.g., with NO_2_, H_2_O etc.) or an electron donor (resistance decrease e.g., with NH_3_, ethanol, acetone and so on). Moreover, the interactions between graphene sheets and the adsorbates could vary from weak Van der Waals to strong covalent bonds [51]. Thus, all these interactions change the electronic structure of graphene, which can be readily monitored by suitable electronic methods (such as through chemoresistors, silicon-based field-effect transistors (FET), capacitance sensors (CS), optical fiber sensors (OFS), and so on [25,26]). In this section, we will deeply discuss pristine graphene combined to metal oxide nanomaterials to be used as promising chemoresistors for the detection of different VOCs, such as ethanol, acetone and several aromatic compounds. In fact, one of the main drawbacks of MOS nanoparticles is still their poor sensitivity and, above all, selectivity. It has been widely reported that hierarchical and fractal structures [52,53] do enhance the gas sensing performances; nevertheless, there is still some room for improvement. A general overview of the most recent results concerning pristine graphene–metal oxides hybrid materials is reported in Table 2 and fully discussed in the following.

Liang et al. [54] described a simple and straightforward method for anchoring α-Fe_2_O_3_ nanoparticles on graphene substrate, tailoring the G content in the MOS matrix. In particular, the α-Fe_2_O_3_@(2 wt%) graphene exhibited a three-fold higher response to 1000 ppm of ethanol than the pure iron oxide material, when operating at 280 °C. In both cases, very short response and recovery times within seconds were recorded, resulting in very useful chemoresistors for practical applications. The authors ascribed this sensing enhancement to several reasons: (i) the high surface area in a 2D network that may facilitate the diffusion of ethanol molecules, thus improving the reaction with surface adsorbed oxygen, namely ionosorption model [39,51,54]; (ii) the material porosity of the hybrid compound with respect to the compact pure Fe_2_O_3_; (iii) the graphene outstanding electrical conductivity resulting in quick electron spreading in the semiconductor surface, showing faster response and recovery times; (iv) finally, graphene might create a Schottky contact at the interface with MOS nanoparticles, favoring α-Fe_2_O_3_ to G electron migration. As such, an enhancement of the sensing performances was attained. Always considering alcohols as target molecules, Qin et al. [55] reported a promising WO_3_-graphene composite prepared through a facile three-step synthetic route comprising the formation of SrWO_4_/graphene oxide (GO) precursor, its transformation into WO_3_/GO by acidification, and the further photoreduction to WO_3_/graphene through UV irradiations. After depositing the as-obtained powder on an alumina tube with gold electrodes, the nanocomposite was tested for different ppm concentrations of alcohols at operating temperatures from 317 to 372 °C. They clearly evidenced that the gas response of the WO_3_/G nanocomposites is considerably better than those relative to pure WO_3_ and WO_3_/GO. Moreover, WO_3_/G did not show any responses to interfering species such as acetone and gasoline, resulting in a promising selective chemoresistor. Its enhanced features may be ascribable to two factors: the improved conductivity related to the presence of graphene, which is highly conductive and therefore it leads to a low signal noise; secondly, since graphene formed parallel layers, the gas diffusion can occur through channels of its slit-shaped pores, providing more active sites for the sensing reaction.

**Figure 1:**
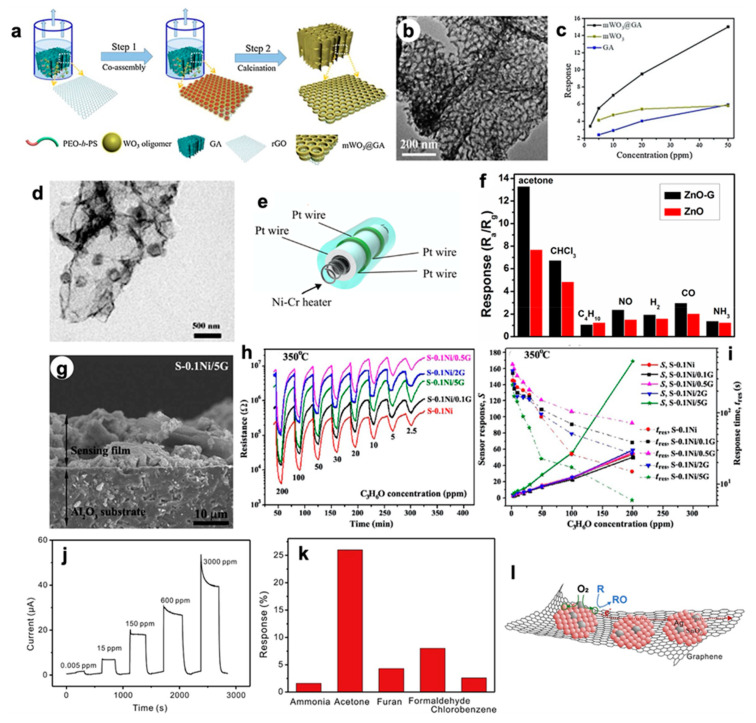
(**a**) Schematic formation process of the mesoporous WO_3_/GA nanocomposites. (**b**) Transmission electron microscopy (TEM) image of mWO_3_@GA. (**c**) Response of the sensors with the increasing of acetone concentrations, at 150 °C. Reproduced with permission from [56]. Elsevier, 2019. (**d**) TEM image of ZnO/graphene nanocomposite [57]. (**e**) Sketch of the prepared sensor device [57]. (**f**) Sensing response of the ZnO/graphene sensor to 100 ppm of different gases at 280 °C [57]. (**g**) Cross-sectional SEM image of S-0.1Ni/5G sensing film (as representative example) on Au/Al_2_O_3_ substrate. (**h**) Change in resistance and (**i**) correlation of sensor response (solid line, left axis) and response time (dash line, right axis) versus acetone concentration of S-0.1Ni/0.1G to 5G sensors, at 350 °C. Reproduced with permission from [58]. American Chemical Society, 2015. (**j**) Dynamic response obtained with Ag/SnO_2_/graphene nanocomposite towards different concentrations of acetone (at 300 °C). (**k**) Comparison of Ag/SnO_2_/graphene sensor responses towards different gases at 150 ppm (300 °C). (**l**) Proposed sensing mechanism of the Ag/SnO_2_/graphene nanocomposite. Reproduced with permission from [59]. Elsevier, 2015.

Tungsten trioxide material was also adopted by Zhao et al. [56] for the synthesis of graphene-based mesoporous composite aerogel for the selective sensing of acetone molecules, at 150 °C. Indeed, through a facile interface-induced co-assembly process (see Figure 1a), mesoporous WO_3_@graphene (mWO_3_@GA) was prepared, in which graphene aerogel (GA) was adopted as a macroporous substrate. 

Mesoporous WO_3_ nanoparticles were homogenously coated on both sides of graphene sheets through a solvent evaporation-induced self-assembly method using a diblock-copolymer as a templating agent. The resultant nanocomposites possess well-interconnected graphene networks covered by mesoporous WO_3_ layer (see transmission electron microscopy, TEM image Figure 1b) with a high surface area of ca. 167 m^2^ g^−1^ and large pores volume of 0.26 cm^3^ g^−1^. Figure 1c shows a comparison of the responses versus acetone concentration variation for pure mWO_3_, GA and the composite mWO_3_@GA. Notably, either the response (within 12 s) or the recovery (ca. 70 s) curves are very sharp denoting quick sensing response, good sensor reversibility and stability. In addition, the signal intensity is much greater for the hybrid mWO_3_@GA, thus demonstrating that it is the best performing material towards acetone molecules with respect to other VOCs. This improvement is stated to be mainly due to the structure and composition of the chemoresistor. In the mWO_3_@GA composite, the graphene aerogel forms 2D graphene sheets that are highly conductive, boosting both the electron transfer efficiency and the electron transfer rate. In addition, the high specific surface area and the interconnected regular mesopores would be beneficial to the surface acetone adsorption/desorption, improving the sensing recovery features. Moreover, in the hybrid structure, many additional active sites, such as oxygen vacancies, can be present, thus enhancing the sensing properties. Zhang et al. [57] investigated another metal oxide matrix, i.e., ZnO combined with graphene, that was revealed as being highly selective to acetone molecules. In particular, by adopting a simple hydrothermal route, they succeeded in decorating the graphene sheets with ZnO nanoparticles, as clearly visible in Figure 1d. The fabricated sensor (displayed in Figure 1e) showed an enhanced response (R_a_/R_g_ of about 13, where R_a_ and R_g_ are the material resistances in air and with the target gas) to 100 ppm acetone, at the optimal operating temperature of 280 °C to two-times higher with respect to that of pure zinc oxide. Furthermore, Figure 1f exhibits the significant selectivity of the nanocomposite towards acetone molecules rather than other organic (as chloroform) or inorganic (such as NO or H_2_) species. For the improved sensing performances, the authors hypothesized that the key role is played by, once more, both the very high graphene specific surface area (of 2600 m^2^ g^−1^) that could lead to a greater amount of surface active sites for the gas adsorption, and the enhanced electron transfer thanks to the presence of graphene material.

As previously touched on, hierarchical and fractal structures do enhance the sensing behavior. In this context, the importance of design chemoresistors with ad hoc features for improved VOC sensing was deeply dealt with by Singkammo et al. [58]. Indeed, in their work, an innovative synthetic approach was exploited, i.e., the flame-spray pyrolysis. The as-prepared 0.1 wt% nickel-doped SnO_2_ nanopowders were loaded with different amounts of graphene to unveil the optimal ratio. The presence of G smoothly enhanced the specific surface area since, with its increase, the surface parameter proportionally rose from ca. 100 to 112 m^2^ g^−1^. Then, the powders were deposited by spin coating on Au/Al_2_O_3_ substrates to evaluate their sensing properties towards acetone molecules. The obtained sensing films showed a significant high thickness of around 12–18 μm (as reported in Figure 1g). Moreover, sensing tests revealed that the addition of 5 wt% graphene led to a dramatical enhancement of the response (a two-order resistance decrease, violet line in Figure 1h), along with a very short response time < 10 s (Figure 1i) towards 200 ppm of acetone (at 350 °C). Once more, the improvement was thought to be due to both the greater specific surface area, achieved thanks to the flame-spray method, and the high electronic conductivity of graphene.

Together with the tailoring of the materials morphology and structure, one of the proposed methods to enhance the selectivity is the nanocomposites decoration with noble metal nanoparticles [65,66]. In this respect, Ge et al. [59] enhanced the selectivity towards acetone of their SnO_2_/graphene nanocomposites by adding silver metal nanoparticles. This novel ternary hybrid compound could sense very low concentrations of acetone, namely 5 ppb (Figure 1j), and its selectivity towards this species was corroborated (Figure 1k). The authors hypothesized that the Ag nanoparticles play a key role in catalyzing the reaction with the target molecules (Figure 1l). Specifically, during the sensing process, the oxygen molecules react preferentially with Ag nanoparticles forming oxygen anions and, then, spill over to SnO_2_ matrix. Reducing species, such as acetone, can be adsorbed onto the surface of silver and then migrate to the surface of tin dioxide, reacting with surface oxygen species and releasing electrons to the nanocomposites. These electrons will be transported by graphene increasing the final conductivity.

**Figure 2:**
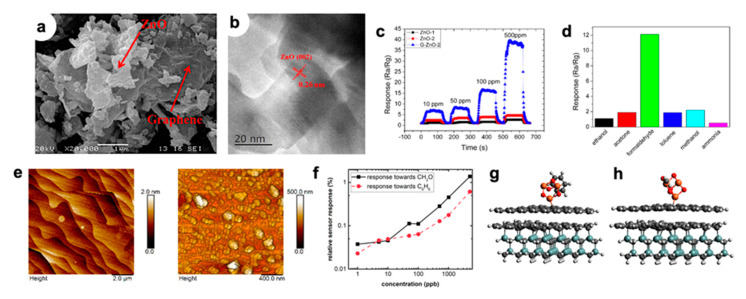
(**a**) Scanning electron microscopy (SEM) and (**b**) high-resolution (HR)-TEM images of ZnO/graphene nanocomposite [60]. (**c**) Response–recovery time curves of pure ZnO-1 (from zinc acetate), pure ZnO-2 (from zinc nitrate), and G-ZnO-2 sensors in the concentration range of 10–500 ppm (at 200 °C) [60]. (**d**) Comparison of signal intensities obtained with G-ZnO-2 towards 100 ppm of different gaseous species [60]. (**e**) Atomic force microscopy (AFM) images of pure epitaxially grown graphene (EG) and Fe_3_O_4_/EG [61]. (**f**) Fe_3_O_4_/EG sensor responses towards different concentrations of formaldehyde (black dots) and benzene (red dots), at 150 °C [61]. Optimized density functional theory adsorption configuration of (**g**) benzene and (**h**) formaldehyde on Fe_3_O_4_/EG [61].

Along with acetone, another investigated VOC for medical diagnosis and environmental concerns is formaldehyde [67]. Hence, Chen et al. [60] deeply studied the enhanced sensing properties of ZnO nanosheets modified with graphene material. By adopting a simple hydrothermal method, they successfully prepared the nanocomposites (G-ZnO-2), as clearly observable in the scanning electron microscopy (SEM) and high-resolution (HR)-TEM images in Figure 2a,b. The sensing measurements indicated that the hybrid material have unique properties as well as good sensitivity, reaching 10 ppm of detected formaldehyde (Figure 2c), optimal selectivity (Figure 2d) and fast response/recovery times of about 10–30 s. Once more, the graphene presence seems to be fundamental for the chemoresistors sensing improvement. In Rodner’s et al. work [61], the authors addressed both the detection of low gases amount and fast response/recovery thanks to the adoption of epitaxially grown graphene that was decorated with iron oxide nanoparticles. Atomic force microscopy (AFM) images clearly exhibit the presence of Fe_3_O_4_ nanoparticles with an average diameter of about 80 nm and an increase in graphene surface roughness (Figure 2e). This hybrid material revealed very sensitive towards formaldehyde and benzene at very low concentrations of parts per billion (see Figure 2f). In addition, the performance enhancement was validated using density functional theory (DFT) calculations to see the effect of decoration on binding energies between the gas molecules and the sensor surface.

**Figure 3:**
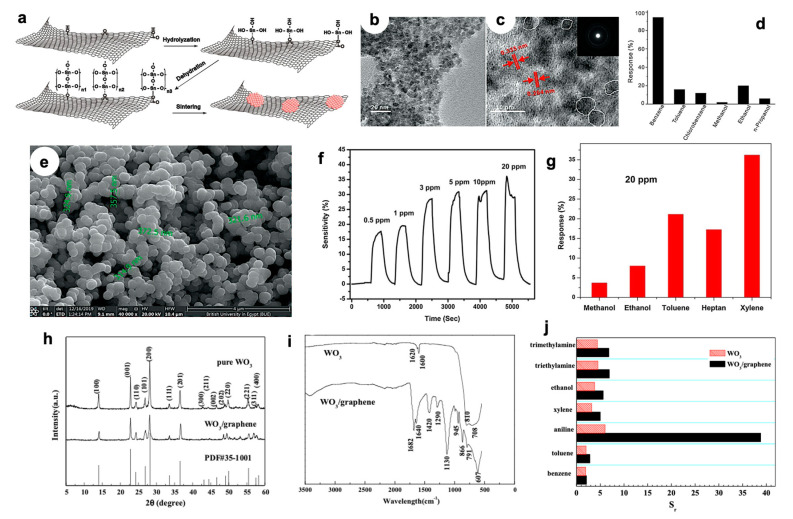
(**a**) The formation mechanism of SnO_2_/graphene nanocomposite materials. (**b**) TEM images of SnO_2_/graphene and (**c**) lattice-resolved HRTEM with corresponding Selected Area Electron Difrraction pattern. (**d**) Comparison of SnO_2_/graphene responses towards 100 ppb of different VOCs (at 210 °C). Reproduced with permission from [62]. Elsevier, 2012. (**e**) Field emission (FE)-SEM micrographs of Co_3_O_4_/graphene composite. **(f**) Dynamic response of Co_3_O_4_/graphene sensor to different concentrations of xylene at RT. (**g**) Selectivity of Co_3_O_4_/graphene material to 20 ppm of different VOCs. Reproduced with permission from [63]. Elsevier, 2020. (**h**) XRD pattern and (**i**) FTIR spectra of bare WO_3_ and WO_3_/graphene samples [64]. (**j**) Comparison of the sensors responses towards various gases at 80 °C [64].

Both benzene and formaldehyde have strong interactions with iron oxide-decorated graphene surface. Specifically, the phenyl ring forms strong bonds in a tilted geometry (Figure 2g), whereas the energetically favored configuration for formaldehyde is through the oxygen atom, giving rise to a strong Fe–O covalent bond with a length of 0.198 nm (Figure 2h). Hence, the authors theoretically demonstrated that either of the two VOC molecules are likely to bind onto the Fe_3_O_4_/EG surface, corroborating their experimental findings. Concerning only benzene detection, several studies showing materials selectivity to this species have been reported so far. For instance, Meng et al. [62] investigated the sensing properties of SnO_2_/graphene nanocomposite synthesized by a simple wet chemical method (see Figure 3a) composed of ca. 5 nm SnO_2_ nanoparticles, as clearly observable in TEM and HRTEM images of Figure 3b,c. In particular, the hybrid material exhibited a much-improved sensitivity compared to bare tin dioxide matrix. Indeed, the achieved detection limit was as low as 5 ppb. In addition, owing to the π-conjugate system of graphene, the response of the nanocomposite to benzene was higher than those towards other VOCs (Figure 3d).

Interestingly, Morsy et al. [63] synthesized a hybrid material comprising of cobalt oxide nanoparticles decorating graphene sheets via a simple impregnation method. Field emission (FE)-SEM revealed the presence of spherical CO_3_O_4_ nanoparticles, which fully cover both the outer and inner pores surface of graphene (Figure 3e), characterized by a specific surface area of around 540 m^2^ g^−1^. The as-prepared sensor showed a good response towards xylene down to 500 ppb at room temperature and a promising selectivity towards this aromatic compound with respect to other VOCs (Figure 3f,g). The authors proposed a mechanism in which CO_3_O_4_ behaves as a p-type semiconductor, resulting in holes as the main charge carriers. When the sensor is exposed to air, O_2_ molecules are adsorbed on the CO_3_O_4_ surface and trap electrons from the conduction band to form oxygen species, namely O_2_^−^, O^−^ and O^2−^. This species formation provokes an increase in the sensor conductivity. Once xylene molecules are fluxed into the sensing chamber, they interact with the surface-chemisorbed oxygen species and release electrons back into the conduction band of CO_3_O_4_, which reduces the hole concentration of the surface charge layer and promotes the resistance of the sensor, according to the following reaction:

2C_8_H_10_ + 21O_2_^−^ → 16CO_2_ + 10H_2_O + 21e^−^ (1)

Finally, regarding the detection of amino compounds, Gui et al. [64] prepared flower-like WO_3_/graphene nanocomposites through microwave-assisted hydrothermal method that exhibited promising sensing features towards aniline molecules at the low temperature of 80 °C. Figure 3h shows the XRD patterns of WO_3_ and WO_3_/graphene nanocomposites displaying the characteristic peaks of the hexagonal WO_3_. This indicates that the addition of graphene does not change the main WO_3_ phase. Any peak of graphene was not observed due to the extremely small amount of this compound used in the nanocomposite. Besides, FTIR spectra of Figure 3i show a broad absorption band at 3400–3500 cm^−1^ associated with the stretching vibration of the –OH functional groups. The absorption peaks at 1682, 1640, 1420, 1130 cm^−1^ in the WO_3_/graphene composite can be ascribable to the vibration of C=O, C=C, C–OH and C–O–C functional groups, respectively. Compared with the pure WO_3_, an additional peak at 1290 cm^−1^ in the nanocomposite provides reliable evidences of a new bond between the graphene and tungsten trioxide. The so-prepared hybrid sample was revealed as being highly sensitive and selective to aniline molecules, as clearly observable in Figure 3j, thanks to the synergistic effect between metal oxide and graphene materials.

## 3. Graphene Oxide-MOS Gas Sensors

Along with the exploitation of pristine graphene, another method recently explored is the coupling of the metal oxides with graphene oxide (GO) [26,41]. Due to its lower defective structure and many surface oxygen-containing functional groups if compared to RGO, graphene oxide has not been studied as much up to now [68,69,70]. Nevertheless, GO was reported to possess excellent electrical and mechanical properties [49] and its p-type semiconductor character [71,72,73,74] can be exploited to form a p-n heterojunction with various n-type gas sensing semiconductors. Moreover, GO functionalities can be the anchor points for the controlled chemical growth of metal oxides, thus giving rise to well-integrated composite materials. Hence, in this section, we will review the main steps forward in the engineering of novel promising chemoresistors comprising the use of graphene oxide, whose performances in terms of detection limit, signal intensity, response and recovery times are listed in Table 3.

**Figure 4:**
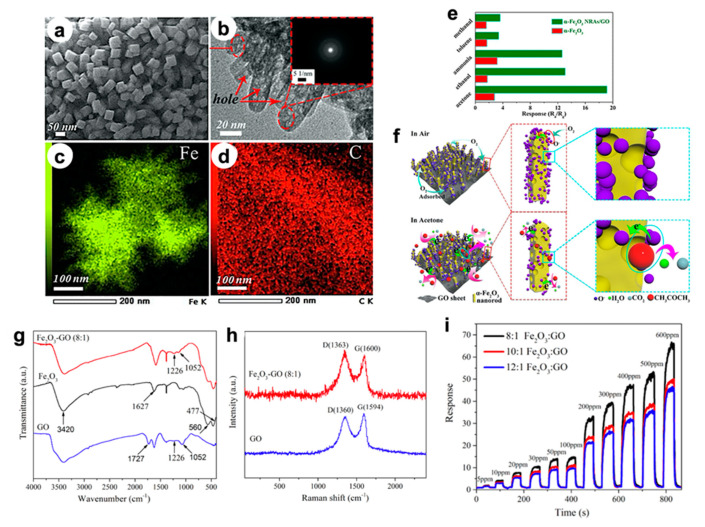
(**a**) SEM and (**b**) TEM images of the α-Fe_2_O_3_ nanorods array (NRAs)/GO hybrid. Energy Dispersive X-ray spectroscopy (EDX) elemental mapping images of (**c**) Fe and (**d**) C species, respectively. (**e**) Response of α-Fe_2_O_3_ NRAs/GO and α-Fe_2_O_3_ nanoparticle-based sensors toward 50 ppm of different gases, at 220 °C. (**f**) Schematic illustration of the gas sensing mechanism. Reproduced with permission from [75]. Elsevier, 2019. (**g**) FTIR spectra of GO, Fe_2_O_3_, Fe_2_O_3_–GO (8:1) nanocomposites [76]. (**h**) Raman spectra of GO and Fe_2_O_3_–GO (8:1) samples [76]. (**i**) Response/recovery curves of Fe_2_O_3_–GO (8:1), Fe_2_O_3_–GO (10:1), Fe_2_O_3_–GO (12:1) nanocomposites towards 5–800 ppm ethanol, at 260 °C [76].

In particular, Song et al. [75] developed an in situ fluorine directed solution process combined with heat treatment to grow highly ordered, porous α-Fe_2_O_3_ nanorod arrays (NRAs) on graphene oxide (GO) sheets, resulting in flexible 3D nanostructures. High-magnification SEM and TEM images (Figure 4a,b) display that both sides of GO sheets were fully covered by vertical nanorod arrays (~50 nm long and 10–20 nm wide), including the edges and wrinkle parts (see EDX analyses in Figure 4c,d). Remarkably, they observed nanopores inside the rods with sizes below 5 nm, which are believed to be beneficial for gas sensing. When tested with 50 ppm of acetone, the nanocomposite showed a high sensitivity at 220 °C, i.e., five times greater than that of pristine α-Fe_2_O_3_ (Figure 4e). Outstandingly, even after 50 days, a stable sensitivity was observed. Moreover, several target molecules were tested, resulting in a slightly higher selectivity towards acetone molecules. Therefore, the improved sensitivity, fast response/recovery and selectivity of the hybrid Fe_2_O_3_ NRAs/GO was attributed by the authors to both the highly ordered, porous structure and the synergistic effect between α-Fe_2_O_3_ and GO. Figure 4f reports a sketch of the possible sensing mechanism. At first, oxygen molecules adsorb on the α-Fe_2_O_3_ NRAs and form O^−^ species at the operating temperature of 220 °C. Then, acetone molecules react with O^−^ species, inducing a material resistance variation, which is recorded by the sensing device. 

In addition, in Jia’s et al. work [76], the authors investigated the same nanocomposite system based on Fe_2_O_3_ grown onto GO via one-step low temperature hydrothermal method. Both FTIR and Raman spectroscopies confirmed the effective accomplishment of the GO decoration by iron oxide nanoparticles. In particular, infrared spectra of Fe_2_O_3_–GO (8:1) reported in Figure 4g show the characteristic band GO, such as C–OH stretching vibrations at 1226 cm^−1^ and C–O–C (epoxy) stretching modes at 1052 cm^−1^; furthermore, the Fe–O vibrational absorption band at 560 and 477 cm^−1^ are clearly observable. In addition, Raman spectra (Figure 4h) exhibit two characteristic peaks relative to D (~1360 cm^−1^) and G (~1594 cm^−1^) bands ascribable to the breathing mode of the *k*-point photons of A_1g_ symmetry and the E_2g_ vibration mode in GO. Notably, for the Fe_2_O_3_–GO (8:1), the relative D/G intensity ratio (ca. 1.07) is slightly higher than that of GO (0.94), confirming the more disordered structure of the nanocomposite, which may be due to the insertion of iron oxide nanoparticles into the GO nanosheets. Finally, the specific surface area of Fe_2_O_3_–GO (115 m^2^ g^−1^) was around three times greater than that of pure iron oxide. Figure 4i displays the dynamic response of the nanocomposites with different Fe_2_O_3_–GO ratios towards ethanol (at 260 °C), resulting in 8:1 the best sensing material. The authors suggested that the improved performances are strictly connected, once more, to the larger surface area and incremental active sites. Hence, the results reveal that GO is crucial to improve the final gas sensing.

**Figure 5:**
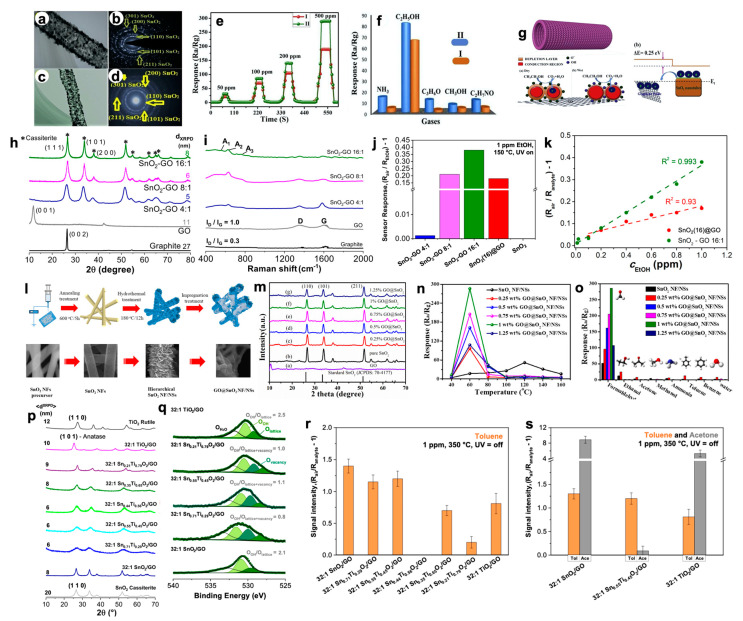
HRTEM of (**a**) pristine SnO_2_ (I) and (**c**) GO–SnO_2_ (II) nanotubes (NTs) along with relative (**b**,**d**) SAED images. (**e**) Dynamic response of pristine and GO–SnO_2_ NTs sensors towards 50–500 ppm of at 300 °C. (**f**) Comparison of sensing response to 100 ppm concentration of various gases at 300 °C. (**g**) Ethanol gas sensing mechanism of GO incorporated SnO_2_ NTs in different atmospheres. Reproduced with permission from [77]. Elsevier, 2019. Structural properties by (**h**) XRPD and (**i**) Raman spectroscopies results relative to graphite, graphene oxide and hybrid SnO_2_–GO samples. (**j**) Comparison of response intensities towards 1 ppm of ethanol by adopting hybrid SnO_2_–GO 4:1, 8:1 and 16:1. For comparison, gas responses acquired with pristine SnO_2_ and SnO_2_(16)@GO (physically prepared by mixing the two powders) samples were reported. Tests carried out in simulated air (80% N_2_–20% O_2_) at 150 °C, under UV light. (**k**) Linear correlation between sensors responses and ethanol concentration for mechanically prepared SnO_2_(16)@GO and SnO_2_–GO 16:1 compounds. Reproduced with permission from [70]. Elsevier, 2019. (**l**) Sketch of the preparation process of GO@SnO_2_ nanofiber (NF)/nanosheets (NSs) [78]. (**m**) XRD patterns of GO, SnO_2_ NF/NSs and GO@SnO_2_ NF/NSs nanocomposites with different GO concentrations [78]. (**n**) Sensing responses toward 100 ppm of formaldehyde at different operating temperatures [78]. (**o**) Response of the gas sensors to different test gases at their respective optimal temperature [78]. (**p**) Comparison of XRD patterns by increasing the Ti content into 32:1 Sn*_x_*Ti_1−*x*_O_2_/GO materials [80]. (**q**) XP spectra of O 1s region relative to three 32:1 Sn*_x_*Ti_1−*x*_O_2_/GO (with *x* = 0.71, 0.55 and 0.21) as representative samples. Additionally, 32:1 SnO_2_/GO and TiO_2_/GO were reported for comparison [80]. (**r**) Comparison of the response intensity of the solid solutions towards 1 ppm of toluene analyte [80]. (**s**) Comparison of the intensities at 1 ppm of toluene (orange) and acetone (grey) obtained with the most promising 32:1 SnO_2_/GO, 32:1 TiO_2_/GO and 32:1 Sn_0.55_Ti_0.45_O_2_/GO samples [80].

For what concerns the exploitation of tin dioxide material coupled with GO, several novel papers have recently been published. For instance, Seshendra Reddy et al. [77] prepared pure SnO_2_ using the one-step electrospinning technique and GO–SnO_2_ nanotubes (NTs) by dipping SnO_2_ NTs into a GO solution followed by a suitable annealing treatment. HRTEM images along with the relative Selected Area Electron Diffraction maps of the hybrid sample revealed the effective GO wrapping on the surface of the SnO_2_ NTs (Figure 5a–d). 

Moreover, in their work, the influence of GO on the ethanol vapor detecting performance of SnO_2_ NTs was analyzed in both dry and wet atmospheres. Figure 5e displays that the GO–SnO_2_ nanotubes showed good response (R_a_/R_g_ of ca. 85) towards 100 ppm of ethanol with an optimum working temperature of 300 °C in dry atmosphere. A slight decrease in the sensor signal (ca. 52) was obtained in wet atmosphere, performing better than pristine tin dioxide nanotubes. Furthermore, it is very selective towards ethanol with respect to other gaseous species (Figure 5f). The authors hypothesized the possible mechanism (displayed in Figure 5g), highlighting the role played by GO. Indeed, the graphene layer attached to the SnO_2_ NTs is believed to enhance the surface area and provide more chemisorbed oxygen on the material surface. In addition, GO stops the agglomeration of tin dioxide nanotubes, guaranteeing a higher active surface area. This could have led to significantly enhanced gas sensing properties. A deeper investigation of the coverage of GO by tin dioxide nanoparticles and the relative ethanol sensing performances were recently evaluated by Pargoletti et al. [70]. The authors of this paper finely investigated different SnO_2_–GO ratios from 4:1 to 16:1 (by a simple hydrothermal method) from different physico-chemical points of view. The structural analyses reported in Figure 5h,i exhibit the effective gradual coverage of GO sheets by SnO_2_ nanoparticles. Moreover, sensing tests towards ethanol species showed that 16:1 ratio is the optimal one in terms of sensitivity, higher signals intensity, good response linearity by reducing the VOC concentration (Figure 5j,k), even with respect to mechanically prepared SnO_2_–GO, namely SnO_2_(16)@GO, and detection at lower operating temperatures (i.e., 150 °C, by exploiting the UV light).

In addition, Wan et al. [78] reported a formaldehyde gas sensor with highly sensitive and selective gas sensing performances at low temperatures based on graphene oxide (GO)@SnO_2_ nanofiber/nanosheets (NF/NSs) synthesized by electrospinning technique followed by a hydrothermal step (Figure 5l). Figure 5m shows the XRD patterns of pristine SnO_2_ NF/NSs, GO@SnO_2_ NF/NSs nanocomposites and GO. Notably, the diffraction peaks of tin dioxide do not change after introducing GO nanosheets, whereas the main diffraction peaks of GO are not perceivable due to its low loading. Indeed, the sensing performances of GO@SnO_2_ NF/NSs nanocomposites was optimized by adjusting the loading amount of GO, ranging from 0.25% to 1.25%. The results show the optimum loading amount of 1% not only exhibited the highest sensitivity value, but also lowered the optimum operation temperature from 120 °C to 60 °C (Figure 5n). Moreover, the GO@SnO_2_ NF/NSs showed a lower detection limit of 0.25 ppm and an excellent selectivity (Figure 5o). The enhanced sensing performances was believed to be mainly due to the high specific surface area, suitable electron transfer channels and the synergistic effect of the SnO_2_ NF/NSs and GO nanosheets network.

Finally, in Kalidoss’s et al. work [79], a ternary composite based on SnO_2_–TiO_2_–GO materials was reported. The gas sensing properties of the as-prepared GO–SnO_2_–TiO_2_ nanocomposites films were investigated at the optimal temperature of 200 °C. The ternary compound exhibited superior gas sensing performances towards acetone in the range 0.25–30 ppm and was also an excellent candidate for the selective detection of this analyte in diabetes mellitus breath. As a step forward from this work, Pargoletti et al. [80] deeply investigated the coupling of SnO_2_–TiO_2_ solid solutions with GO (with 32:1 solid solution/GO weight ratio) to unravel the possible boosted selectivity of the prepared compounds towards aromatic species. A fine tuning of the Ti content into a SnO_2_ matrix was obtained, as clearly represented in the XRD patterns, resulting in an increased crystallinity due to a rise in particle size attained at high Ti content, i.e., at the phase transition from cassiterite-like to rutile-like crystal structure (Figure 5p). Remarkably, with the increase in Ti amount, a higher lattice defectivity was seen, corroborated by X-ray photoelectron spectroscopy (XPS) analyses. Indeed, focusing on the O 1s region (Figure 5q), the band relative to loss of oxygen or creation of oxygen vacancies is visible in the 32:1 Sn*_x_*Ti_1−*x*_O_2_/GO nanocomposites. Notably, these compounds were revealed to be more sensitive and selective towards toluene molecules at 350 °C (Figure 5r,s). The authors hypothesized that a low titanium content limits the cross-sensitivity to relative humidity by reducing the number of rooted and terminal hydroxyl surface species with respect to both pure metal oxides, thus becoming less hydrophilic and, hence, more prone to sense non-polar molecules as toluene ones.

**Figure 6:**
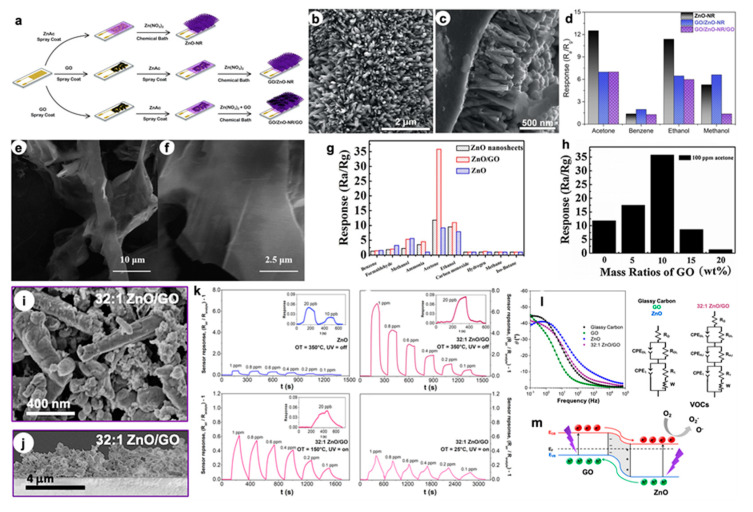
(**a**) Synthetic route used to prepare ZnO-NR, GO/ZnO-NR and GO/ZnO-NR/GO samples. SEM image of (**b**) GO/ZnO-NR and (**c**) cross-section micrograph of the ZnO-NR arrays. (**d**) Comparison of the responses of ZnO-NR, GO/ZnO-NR, and GO/ZnO-NR/GO to 200 ppm of different VOCs (at 450 °C). Reproduced with permission from [81]. Elsevier, 2016. (**e**,**f**) FESEM images of ZnO/GO nanocomposites. (**g**) Sensing responses of pure ZnO, ZnO nanosheets and ZnO/GO nanocomposites sensors to 100 ppm of different gases, at 240 °C. (**h**) Responses obtained with different GO mass ratios in the ZnO/GO nanocomposites (100 ppm acetone, at 240 °C). Reproduced with permission from [82]. Elsevier, 2016. SEM image of (**i**) 32:1 ZnO/GO and (**j**) relative cross-sections of deposited layers [83]. (**k**) Dynamic responses towards various ethanol concentrations, by decreasing the operating T (at 150 °C and RT, exploiting the UV light) [83]. (**l**) Electrochemical Impedance Spectroscopy (EIS) Bode plots with corresponding fitting circuits [83]. (**m**) Schematic model of GO-ZnO p-n junction for hybrid materials under UV and with +1.0 V applied bias (E_VB_, E_CB_, E_F_ are energies of Valence Band, Conduction Band and Fermi level) [83].

In parallel to the tin dioxide semiconductor, ZnO-based materials have also been widely studied in the gas sensing field. Vessalli et al. [81] recently described an innovative synthetic route to grow ZnO nanorods (ZnO-NR) with controlled size and morphology on gold interdigitated electrodes (IDEs) via chemical bath deposition, at mild temperatures (90 °C; see Figure 6a). 

Furthermore, through the same technique, ZnO-GO compounds were obtained in which graphene oxide sheets can be present below (GO/ZnO-NR) or on both sides of ZnO-NR (GO/ZnO-NR/GO), like a sandwich. The morphology of the as-obtained materials was deeply studied by SEM analysis. Particularly, from the cross-section image in Figure 6c, it is possible to determine the NRs length of about 640 nm. Conversely, the presence of GO in GO/ZnO-NR sample has led to larger diameters (Figure 6b) and the further increase in GO (GO/ZnO-NR/GO compound) caused the coverage of the ZnO surface by the graphene material itself. These structures, then, were tested as sensors for volatile organic compounds such as acetone, benzene, ethanol and methanol in the concentration range of 10–500 ppm, at the optimal operating temperature of 450 °C (Figure 6d). Notably, both the pure ZnO-NR and the GO/ZnO-NR/GO composite showed better selectivity towards acetone molecules, thanks to the either the peculiar morphological features or the presence of GO functional groups that favor the interaction with the target polar VOC. In addition, Wang et al. [82] finely explored the effect of GO content in the ZnO matrix on the final sensing. Specifically, both bare and phosphorus-doped ZnO nanosheets were produced by the CVD method, and GO was introduced by simple impregnation, adopting different graphene oxide amounts. FESEM micrographs reveal the presence of trigon-star like-shaped pure ZnO nanoparticles with branch lengths of around 5 μm that tend to become smooth after P doping (Figure 6e,f). The nanosheets lengths are around tens of micrometers, several micrometers wide and around 50 nm thick. The authors found that 10 wt% GO is the optimal loading to reach very high responses (R_a_/R_g_ of around 36) towards 100 ppm of acetone (Figure 6h). Moreover, this material seems to be the best performing one in terms of selectivity to acetone molecules, as reported in Figure 6g. The composite unique sensing properties are believed to be due to the presence of planar GO sheets, which offer an extensive 3D network, thus enhancing the interconnectivity among the ZnO nanosheets. In addition, defects and functional groups on the GO surface act as high-energy adsorption sites for the gas molecules and they can increase the final response. Nevertheless, for higher GO content (more than 10 wt%), partial ZnO nanosheets are covered by graphene oxide, affecting the active interacting area of ZnO and acetone gas. So far, studies concerning the enhancement of the sensing features mainly at high working temperatures have been reported; however, to be used in portable devices, such chemoresistors should operate at low temperature. In this context, Pargoletti et al. [83] recently described novel ZnO-GO 3D nanonetworks which are able to sense different VOCs at room temperature, by exploiting the UV light. In addition, in the present work, the authors deeply investigated the optimal ZnO/GO ratio in order to get the best sensing performances, resulting in the 32:1 ZnO salt precursor-to-GO weight ratio being the most promising one. By adopting a simple hydrothermal method, the gradual coverage of GO sheets occurred, as clearly observable in Figure 6i, since with the lowest ratio (4:1) the characteristic GO morphology was still visible. Once synthesized, the nanopowders were deposited by an innovative hot-spray method giving rise to micrometric and highly porous (> 90%) films onto IDEs (Figure 6j). Remarkably, only with the 32:1 ZnO/GO material was a successful signal towards different VOCs (such as acetone, ethanol and ethylbenzene) recorded down to RT by exploiting the UV light. Nevertheless, this nanocomposite showed a higher selectivity towards small and polar analytes, such as ethanol, for which 100 ppb were detectable even at RT (see Figure 6k). Therefore, the unique features of these hybrid compounds were finely investigated to understand their sensing mechanism. For this reason, electrochemical impedance spectroscopy (EIS) was adopted to possibly unveil the synergistic effect of GO and n-type semiconductors, like zinc oxide. Figure 6l exhibits the Bode plots of both chemically obtained compounds and mechanically mixed ZnO/GO with the same 32:1 ratio. Only with the 32:1 ZnO/GO was it possible to observe the presence of a third circuit relative to the heterojunction occurrence at the interface between graphene oxide (p-type) and ZnO (n-type) materials. This p-n nano-heterojunction could lead to boosted sensing properties, since an increased electron concentration is present in the conduction band of ZnO, thus favoring a risen amount of negatively charged oxygen species adsorbed onto the sensor surface (Figure 6m). Indeed, these species are believed to be the main protagonists of the sensing mechanism, according to the already described ionosorption model [51,84].

## 4. Reduced Graphene Oxide-Based Chemoresistors

Among the strategies for developing high-performance sensors, the use of reduced graphene oxide (RGO) can improve the final sensing performances, such as increased sensitivity, selectivity, room temperature operating conditions and low cross-sensitivity to humidity (one of the main interfering species in humans’ breath) [25,42,49,85]. In this context, several studies have been carried out, and they are summarized in Table 4.

**Figure 7:**
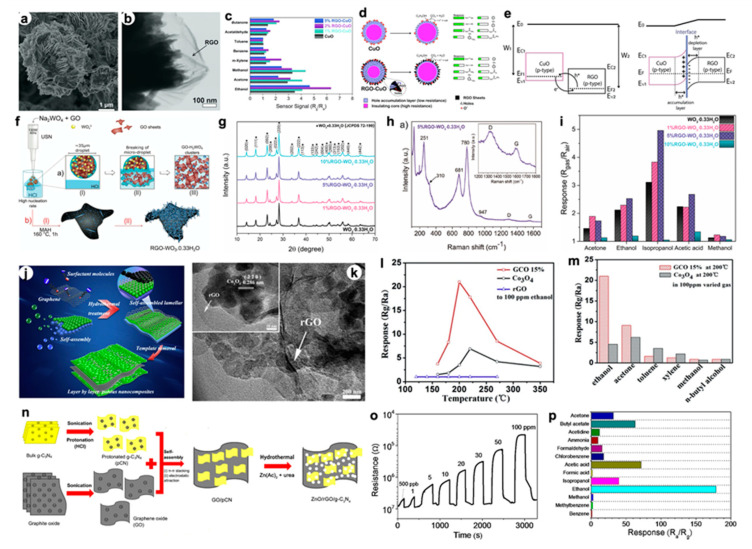
FESEM micrograph of (**a**) 2% RGO–CuO composite and (**b**) relative HRTEM image. (**c**) Sensors signal to 100 ppm of different VOCs at 250 °C, in dry air. (**d**) Scheme of the ethanol sensing mechanism by pure CuO and RGO–CuO. The bars represent the relative response to different VOCs. (**e**) The energy band diagrams of CuO and RGO before creating the junction and for the p-p heterojunction. Reproduced with permission from [86]. Elsevier, 2019. (**f**) Scheme of growth and formation of RGO–WO_3_⋅0.33H_2_O. (**g**) XRD patterns of the pure sample and RGO–RGO–WO_3_⋅0.33H_2_O composites. (**h**) Raman spectra of RGO–WO_3_⋅0.33H_2_O composites. (**i**) Sensing response to 100 ppm of different VOCs. Reproduced with permission from [87]. The Royal Society of Chemistry, 2018. (**j**) Scheme of the adopted synthetic route. (**k**) TEM image of RGO–Co_3_O_4_ (GCO) 15% sample. (**l**) Responses to 100 ppm of ethanol vapor with the variation of the operating temperature. (**m**) Comparison of pure Co_3_O_4_ and GCO 15% responses to various gases. Reproduced with permission from [88]. Elsevier, 2019. (**n**) Synthetic approach used to prepare the ternary ZnO/RGO/g-C_3_N_4_ composites. (**o**) Dynamic response of ZnO/rGO/g-C_3_N_4_ sensor to different concentrations of ethanol at 300 °C. (**p**) Selective test with ZnO/rGO/g-C_3_N_4_ sensor to 100 ppm of different gases. Reproduced with permission from [89]. American Chemical Society, 2019.

For instance, concerning alcohols detection, Modenes-Junior et al. [86] succeeded in fabricating RGO-CuO nanocomposites using one-step microwave-assisted synthesis. FESEM and HRTEM images show the formation of hierarchical CuO spherical particles with a diameter of about 4 μm. Furthermore, for the 2% RGO–CuO composite (Figure 7a,b), the spherical morphology was conserved and RGO sheets cover part of the CuO surface, promoting the interaction between the two materials. Furthermore, the 2% RGO–CuO sensing response towards 100 ppm of different VOCs (acetaldehyde, acetone, benzene, butanone, ethanol, methanol, m-xylene and toluene) at 250 °C was evaluated (Figure 7c).

The nanocomposites with RGO content of 2% and 5% exhibited, respectively, an ethanol response of about 2–6 and 2–5 times higher than that towards other VOCs. These results suggest that the addition of RGO in appropriate contents (2 and 5 wt%) improved the selectivity of the sensors to ethanol. Since the VOC selectivity is directly connected to the receptor function of the MOS surface, the presence of ideal RGO contents might change the reactivity of the material surface, which, consequently, modifies the final sensitivity. Therefore, the enhanced ethanol sensing performance of RGO-CuO was attributed to the RGO/CuO heterojunction. When p-type CuO is exposed to air at the temperature range of 100–500 °C, molecular oxygen adsorbs and ionizes into different species (as mentioned in Section 2). This process creates a hole (h^+^) accumulation layer near the material surface (Figure 7d). When the material is exposed to ethanol, the gas molecules react with the ionosorbed oxygen species, oxidizing ethanol. Thus, electrons are released to CuO, reducing the hole accumulation layer and increasing the insulating core, which enhances the material resistance.

RGO positively contributed to the ethanol sensing since both CuO and reduced graphene oxide are p-type semiconductors, and the combination of these materials creates a p-p heterojunction. Specifically, CuO and RGO have a work function of 5.2 and 4.6 eV, respectively, with a band gap of 1.2–1.9 eV and 0.4 eV [86]. Therefore, being the Fermi energies at different levels, the electrons flow from CuO to RGO, while holes are transferred in the reverse direction. As a result, holes are accumulated near the CuO surface, and a depletion layer is created in RGO (Figure 7d,e). In this way, when ethanol molecules are present, an enhanced resistance variation occurs, resulting in boosted sensing features.

A noteworthy characteristic of graphene-based materials is their flexibility. In this context, Perfecto et al. [87] engineered a flexible sensor device comprising the use of WO_3_⋅0.33H_2_O nanoneedles hybridized with RGO material (at different RGO amounts), deposited onto polyethylene terephthalate (PET) substrate. The materials were synthesized via a combination of the ultrasonic spray nozzle (USN) and microwave-assisted hydrothermal (MAH) methods (Figure 7f) to obtain a single orthorhombic crystalline phase in which RGO presence is not detectable due to its negligible amount (Figure 7g). Moreover, the Raman spectrum (Figure 7h) exhibits the effective growth of WO_3_ nanoneedles onto graphene oxide sheets and the subsequent reduction of this material to RGO. Then, the authors studied the VOC sensing properties of the materials deposited on PET electrodes at room temperature and at 55% of relative humidity (RH). Notably, the 5%RGO–WO_3_⋅0.33H_2_O showed superior isopropanol-selective sensing performances, with an almost two-fold higher response with respect to the bare compound (Figure 7i). Furthermore, Tian et al. [88] prepared a layer-by-layer nanocomposite consisting of Co_3_O_4_ and reduced graphene (RGO) nanosheets by a simple hydrothermal process followed by an annealing step (see scheme in Figure 7j), which was very promising for the selective detection of ethanol molecules (at 200 °C, see Figure 7l). The effective synthesis accomplishment was confirmed by TEM images which show wrinkle-like RGO underneath mesoporous cobalt oxide nanosheets (see Figure 7k). Among the prepared nanocomposites, the 15 wt% RGO exhibited the best sensitivity and selectivity to ethanol analyte (Figure 7m) due to both the heterojunction formation at the interface between the two materials and the increase in defects and vacancies after RGO addition, resulting in the formation of more active sites for gas adsorption and surface reactions. The step forward in the detection of alcohol species stated by Meng et al. [89] consists of the development of a ternary compound comprising the adoption of graphitic carbon nitride (g-C_3_N_4_) nanosheets as a sensitization modifier for zinc oxide (ZnO)/reduced graphene oxide (RGO), rather than the widely studied and already reported noble metal nanoparticles. The nanostructures of 2D GO-hybridized by g-C_3_N_4_ were, at first, synthesized by combining ultrasonic dispersion and electrostatic self-assembly. Then, ZnO nanoparticles were hydrothermally coated on GO/g-C_3_N_4_: in this step, GO was totally reduced to RGO (Figure 7n). XRD patterns clearly exhibit the ZnO wurtzite phase, whereas no characteristic peaks of GO and g-C_3_N_4_ can be observed. Furthermore, compared to pure ZnO and ZnO/RGO, the ZnO/rGO/ g-C_3_N_4_ has a remarkable enhancement in response to ethanol molecules which were sensed down to 500 ppb (at 300 °C), as displayed in Figure 7o,p. The enhancement was attributed to either the sensitization of g-C_3_N_4_ or the extended interfacial contact between RGO and the graphitic carbon nitride.

**Figure 8:**
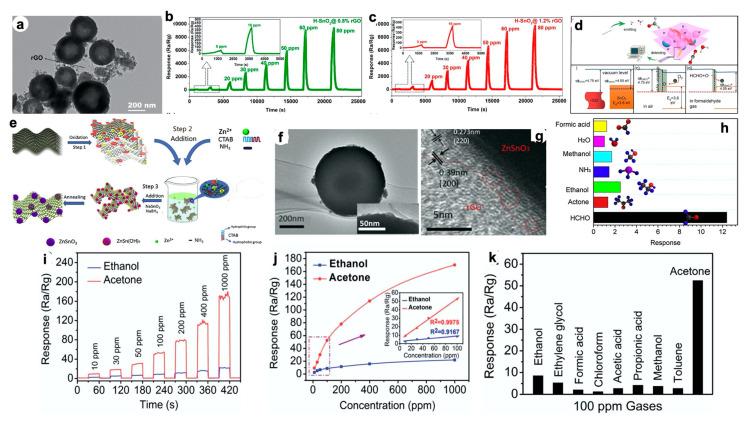
(**a**) TEM image of H-SnO_2_@1.2%RGO. Formaldehyde sensing curves of (**b**) H-SnO_2_@0.8%RGO and (**c**) H-SnO_2_@1.2%rGO composites at 130 °C towards various gas concentrations (5−80 ppm). (**d**) Schematic illustration of the sensing mechanism of H-SnO_2_@RGO sensors with band diagram of the RGO and SnO_2_ before contacting and in H-SnO_2_@RGO composites, either in air or upon formaldehyde flux. Reproduced with permission from [92]. Elsevier, 2019. (**e**) Synthetic route for the fabrication of RGO/ZnSnO_3_ composites. (**f**,**g**) HRTEM images of the 3 wt% rGO/ZnSnO_3_. (**h**) Comparison of sensor responses towards several gaseous species (at ca. 100 °C). Reproduced with permission from [93]. Elsevier, 2017. (**i**) Sensor responses of CuO–ZnO/RGO towards acetone and ethanol analytes (at 340 °C). (**j**) Signal intensity trend with increasing of ethanol and acetone concentrations. (**k**) Comparison of the CuO–ZnO/RGO sensor performances to 100 ppm of various VOCs. Reproduced with permission from [94]. The Royal Society of Chemistry, 2014.

In addition, as regards aldehydes and ketones sensing by RGO-based composites, several investigations have already been carried out and reported [90,91,99]. Hu et al. [92] very recently explored novel reduced graphene oxide-wrapped hollow SnO_2_ nanosphere (H-SnO_2_@RGO) composites successfully synthesized via a simple hydrothermal method, without the addition of any templating agents. Figure 8a confirms the attachment of the flexible graphene sheets to hollow SnO_2_ nanospheres. Moreover, different RGO contents (i.e., 0.8, 1.2, 2.0 and 4.0%) were investigated, resulting in 1.2% being the best performing chemoresistor. Indeed, this material, together with 0.8%, showed very high sensitivity (detection limit, LOD, of 5 ppm), even under a 95% humidity atmosphere, to formaldehyde species at 130 °C (Figure 8b,c). The increased performances are mainly due to the synergistic effect between RGO and SnO_2_. Indeed, as previously reported for Modenes-Junior’s et al. [86] work, also here the sensing mechanism is almost the same (see Figure 8d). The only exception consists of the adoption of an n-type semiconductor (as tin dioxide), instead of a p-type one (as CuO). Specifically, in order to equalize the energy of the SnO_2_ and RGO Fermi levels, electrons tend to go from the conduction band of tin dioxide to that of RGO. As such, due to the formation of negatively charged oxygen species adsorbed on the materials surface, the depletion layer gets wider. Upon purging the reducing VOC, electrons deriving from the reaction are released back to the chemoresistor, thus decreasing the materials resistance. Sun et al. [93] described a new material based on RGO/ZnSnO_3_ composites prepared by a facile solution-based self-assembly synthetic method occurring at low temperature (as represented in Figure 8e). The microtextures of RGO/ZnSnO_3_ were studied by SEM, HRTEM and XRD analyses. In particular, the morphology of ZnSnO_3_ was controlled by the external addition of surfactant and ammonia to form hierarchical microspheres tightly anchored on RGO (Figure 8f,g). Concerning the sensing behavior, the 3 wt% RGO/ZnSnO_3_ was revealed as being very promising for the sensing of formaldehyde, since not only did it show excellent sensitivity (LOD of 1 ppm), selectivity (Figure 8h) and response linearity, but it also demonstrated very fast response and recovery at the operating temperature of 103 °C.

Furthermore, two other research papers are worth noting, both on the design of innovative composites for the selective detection of acetone molecules. The first one, by Wang et al. [94], reported the one-step hydrothermal strategy to synthesize CuO–ZnO/RGO ternary composites with outstanding features (Figure 8i), resulting in a sensing response about 10–60 times greater with respect to other widely investigated VOCs (Figure 8j). The second article was about the engineering of tungsten trioxide/reduced graphene oxide-based materials, which was again very promising for the acetone detection at 22 °C and RH of 55%. Specifically, as already unraveled in a previously cited paper, Perfecto et al. [95] successfully synthesized RGO-h-WO_3_⋅0.33H_2_O (hexagonal WO_3_ polymorph) by the one-pot microwave-assisted hydrothermal method. The authors found that all the materials behave as p-type semiconductors after being exposed to reducing gases, evidencing a 20% greater response for the composite material with respect to pure WO_3_ (Figure 8k).

**Figure 9:**
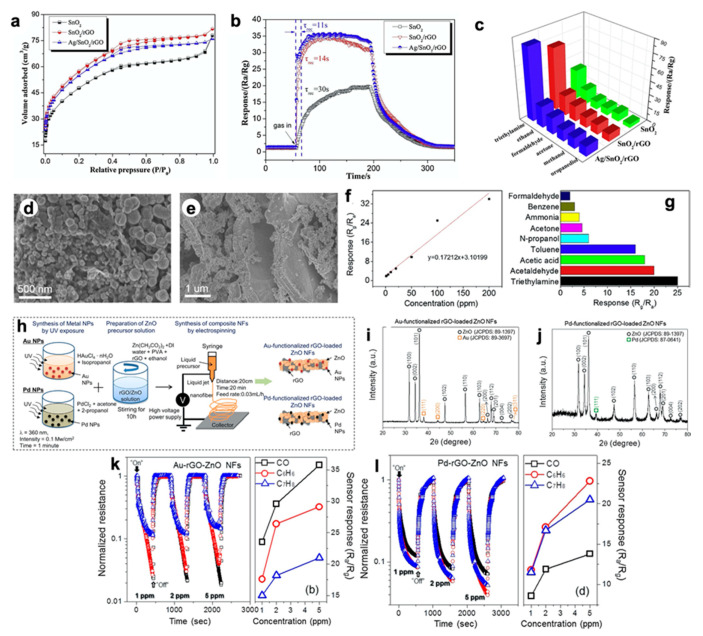
(**a**) BET data of pure SnO_2_, SnO_2_/RGO and Ag/SnO_2_/RGO samples. (**b**) Response transient of the three sensors to 20 ppm of TEA. (**c**) Selectivity of the three materials to 100 ppm of different gases. Reproduced with permission from [94]. Elsevier, 2019. SEM images of (**d**) pure Co_3_O_4_ and (**e**) D-Co_3_O_4_/rGO-2 (with different RGO amounts). (**f**) Linearity of the D-Co_3_O_4_/rGO response versus TEA concentration. (**g**) Selective testing of the D-Co_3_O_4_/RGO sensor at 200 °C. Reproduced with permission from [97]. Elsevier, 2019. (**h**) Schematic illustration of the synthesis for Au- or Pd-functionalized RGO-loaded ZnO nanofibers (NFs). XRD patterns of (**i**) Au- and (**j**) Pd-functionalized RGO-loaded ZnO NFs. Dynamic response of (**k**) Au- and (**l**) Pd-functionalized RGO–ZnO NFs sensor to different concentrations of CO, benzene and toluene gases with the corresponding calibration curves. Reproduced with permission from [98]. Elsevier, 2017.

Additionally, concerning aromatic and amino compounds, a couple of studies should be highlighted. Firstly, Zhang et al. [96] recently presented a ternary compound, namely Ag/SnO_2_/reduced graphene oxide (RGO) fabricated through a one-step route and comprising the decoration with noble metal nanoparticles (i.e., silver ones). The coexisting of tin dioxide and Ag was mainly corroborated by XRD analysis, whereas BET measurements (Figure 9a) evidenced an increase in the active surface area with the addition of RGO (from 171 to 205 m^2^ g^−1^), along with an average pore size distribution centered at around 4 nm.

The novel ternary compound was adopted for the sensing of different gaseous species, resulting in a very fast response (t_res_ of ca. 10 s; Figure 9b) and a great selectivity towards triethylamine (TEA) molecules (at 220 °C; see Figure 9c). This marked enhancement of TEA may be related to both the catalytic activity of silver nanoparticles and the higher specific surface area of the Ag/SnO_2_/rGO nanocomposite. Remarkably, the as-prepared compound revealed a very promising stability over time, i.e., even after 25 days. In addition, Yuan et al. [97] focused their research on the design of a sandwich-like double layer (D) material comprised of Co_3_O_4_ nanoparticles and reduced graphene oxide. Figure 9d,e show SEM images of composite materials synthesized by varying the RGO amount. Notably, D-Co_3_O_4_/RGO-2 (Figure 9e) clearly exhibits a double layer structure, in which the cobalt oxide nanoparticles grew along the sides of RGO sheets resulting in a double layer structure. This sandwich-like composite was tested towards different VOCs (Figure 9g) at its optimal operating temperature of 200 °C. The results indicate that the composites have a five-fold linear increase in the response to 100 ppm of TEA compared to bare Co_3_O_4_ sensor, thanks to the peculiar structure and great active surface area. Moreover, the signal intensity has a linear behavior with the decrease in the sensed TEA concentration (Figure 9f) down to 1 ppm. Finally, Abideen et al. [98] investigated the tailoring of RGO–ZnO functionalization by different noble metal nanoparticles, namely Au and Pd ones. The authors adopted a combination of facile, cost-effective sol-gel and electrospinning methods as illustrated in Figure 9h. The effective accomplishment of the synthetic route was obtained by XRD analyses, whose patterns revealed the presence of gold or palladium in the corresponding nanocomposites (Figure 9i,j). Notably, the Pd-based compound showed a remarkable response towards benzene species, rather than the other tested VOCs. Meanwhile, the presence of gold seems to favor the detection of inorganic gases, such as carbon monoxide (Figure 9k,l). The boosted sensing features are believed to be due to both the presence of RGO/ZnO hetero-interfaces and the catalytic effect of Au and Pd nanoparticles. These results show that the combination of noble metals, such as Au or Pd NPs, with RGO and ZnO can impart new gas sensing functionalities that are potentially useful for the sensing of either inorganic or organic volatile compounds.

## 5. Carbon Nanotubes-Metal Oxides Sensing Materials

The majority of the scientific studies reported so far emphasized the main drawback still to be solved, i.e., the high operating temperatures. In this respect, carbon nanotubes (CNTs) can represent a valuable alternative. Indeed, since the first reports on single-wall carbon nanotubes (SCNTs) by Japanese [100] and American [101] groups around 30 years ago, there has been great interest in exploring the unique electrical, physical, mechanical and chemical properties of both single-wall and multi-wall CNTs in view of developing well-performing devices [102]. Furthermore, their physico-chemical features make them suitable for gas sensing. Specifically, the detection mechanism of bare CNT gas sensors is based on the variation of electrical conductivity after the adsorption of gas molecules. Remarkably, their main advantage is their response even at room temperature, which is optimal for ultra-low power, wearable, miniaturized devices. To boost the final performances, their coupling with MOS nanoparticles can be a solution to reduce the operating temperature while keeping the already promising properties of semiconductor materials. Notwithstanding this, only a few articles about CNTs-MOS composites sensors have been reported up to now [103,104,105] (see Table 5).

**Figure 10:**
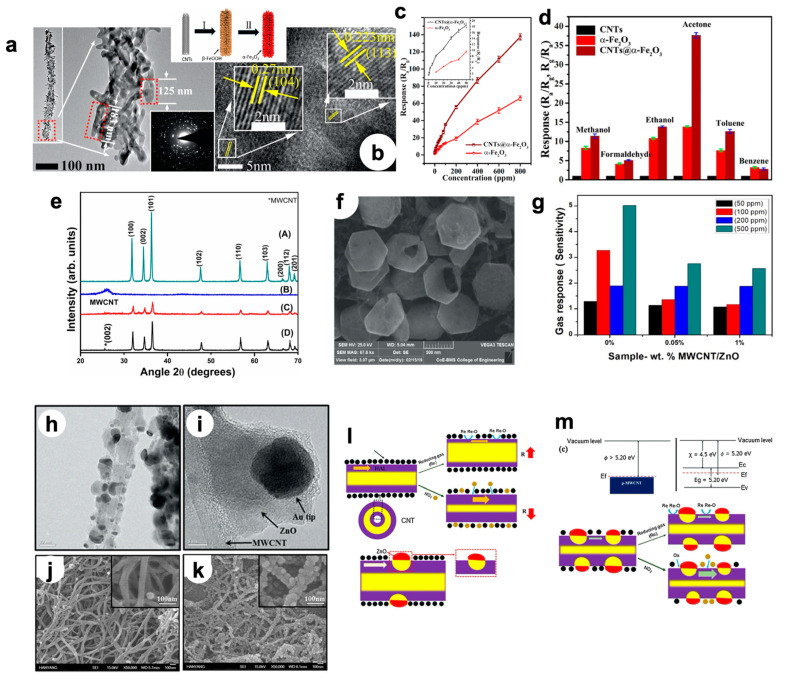
(**a**) TEM image of CNTs@α-Fe_2_O_3_ composites and corresponding SAED pattern in inset; (**b**) HRTEM image of CNTs@α-Fe_2_O_3_ composites. Inset: schematic representation of the formation of α-Fe_2_O_3_ nanorods coated on the CNT surface: (I) heterogeneous growth of β-FeOOH nanospindles via force hydrolysis of Fe^3+^ ions; (II) thermal transformation of β-FeOOH into α-Fe_2_O_3_ nanorods on CNTs via annealing CNTs@FeOOH structures in air. (**c**) Response of the sensors based on the CNTs@α-Fe_2_O_3_ composites and single α-Fe_2_O_3_ versus the acetone concentration at 225 °C. (**d**) Comparison of the response of the CNTs@α-Fe_2_O_3_ composites, single α-Fe_2_O_3_ and CNTs sensors to 100 ppm of various gases at 225 °C. Reproduced with permission from [44]. American Chemical Society, 2017. (**e**) Comparison of XRD spectra of (A) pure ZnO, (B) multi-wall carbon nanotubes (MWCNTs), (C) 1 wt% MWCNT/ZnO and (D) 0.05 wt% MWCNT/ZnO composites. (**f**) SEM image of 1 wt% MWCNT/ZnO composite. (**g**) Comparison of sensing responses towards different concentrations of 1-butanol (at RT). Reproduced with permission from [103]. Elsevier, 2019. (**h**,**i**) TEM and (**j**,**k**) images of MWCNT/ZnO composite. (**l**) Schematic representation of modulation of the Hole Accumulation Layer (HAL) in bare MWCNTs, during the adsorption of oxidizing and reducing gas molecules, and width decrease by the p-n junction between p-MWCNTs and n-ZnO. (**m**) Energy band diagrams of p-MWCNT and n-ZnO and modulation of the HAL in n-ZnO-p-MWCNTs nanocomposite sensor. Reproduced with permission from [110]. Elsevier, 2017.

For instance, concerning the adoption of multi-wall carbon nanotubes (MWCNTs), Wang et al. [106] investigated chemoresistors based on tin oxide doped with hydroxyl (–OH) functionalized MWCNTs for the sensing of indoor formaldehyde. The response of the 5 wt% MWCNTs-doped SnO_2_ sensor was much higher than that of bare tin dioxide material, resulting in 30 ppb, the lowest detectable concentration. In addition, Dai et al. [44] presented a facile hydrolysis reaction (I in inset of Figure 10a,b) followed by annealing step (II) for the synthesis of a novel hierarchical nano-heterostructure via the assembly of α-Fe_2_O_3_ nanorods onto MWCNTs. TEM (Figure 10a,b) images show uniform α-Fe_2_O_3_ nanorods of approximately 100–200 nm in length and 50–100 nm in diameter hierarchically assembled onto the surface of the CNTs.

Moreover, XRD analysis confirmed the presence of both carbon-based material (i.e., (0 0 2) plane at ca. 26.3°) and the hexagonal α-Fe_2_O_3_ (namely hematite polymorph). Notably, the composites exhibited excellent sensing properties for acetone with high sensitivity, remarkable selectivity and good reproducibility (Figure 10c,d), superior than the bare α-Fe_2_O_3_. The boosted sensing properties were mainly attributed to the unique structure and p-n heterojunction between the p-type CNTs and the n-type α-Fe_2_O_3_ nanorods. Furthermore, Bhat et al. [107] explored zinc oxide and multi-wall carbon nanotubes ZnO/MWCNT composites, adopting a wet chemical method. The effective growth of hexagonal wurtzite ZnO on MWCNTs surfaces was corroborated by either XRD analysis (Figure 10e) and SEM micrographs (Figure 10f). In particular, they exhibit the characteristic hexagonal ZnO discs, whereas composite samples are comprised of thin fibers of MWCNTs in between the ZnO nanoparticles. These MWCNTs help in joining the ZnO nanodiscs and improve the conductivity of the final material. The as-prepared sensors showed promising selective features at RT towards 1-butanol molecules (Figure 10g), with a LOD of about 50 ppm.

Kwon et al. [110], instead, unraveled the mechanism behind the sensing behavior of MWCNTs and a zinc oxide semiconductor. Indeed, by in situ thermally evaporating Zn powders in the presence of multi-wall carbon nanotubes, they successfully fabricated p-n nano-heterojunctions. The optimal integration of MWCNTs into the ZnO matrix was confirmed by XRD, TEM (Figure 10h,i) and SEM (Figure 10j,k) images, revealing the concomitant presence of wurtzite ZnO spherical nanoparticles and the elongated morphology of the CNTs. The authors found that the decoration with ZnO nanoparticles greatly improves the gas sensing properties of bare MWCNTs, thanks to the synergistic effect between these two materials. In particular, in the case of bare MWCNTs, they stated that, in air, adsorbed oxygen anions capture electrons form the material surface, thus forming a hole accumulation layer (HAL) that leads them to behave as p-type semiconductors (Figure 10l). When MWCNTs are covered by ZnO nanoparticles, several factors can boost the sensing behavior. First of all, ZnO enlarged the active surface area, facilitating the effective molecule adsorption; secondly, this MOS could provide a spillover effect, favoring the adsorption, dissociation and migration of the target molecules; thirdly, p-n heterojunctions can be formed at the interface (Figure 10l,m). As displayed in Figure 10m, electrons flow from ZnO to MWCNT and holes flow in the opposite direction, until the formed built-in potential hinders the further flux of the charged carriers. Therefore, the width of HAL in the p-MWCNTs is decreased. When a reducing species, such as ethanol or acetone, is introduced into the sensing chamber, the reduction in holes in CNTs by the gas adsorption causes a greater sensor response. Finally, upon their reaction with adsorbed oxygen, the released electrons further decrease the material resistance.

**Figure 11:**
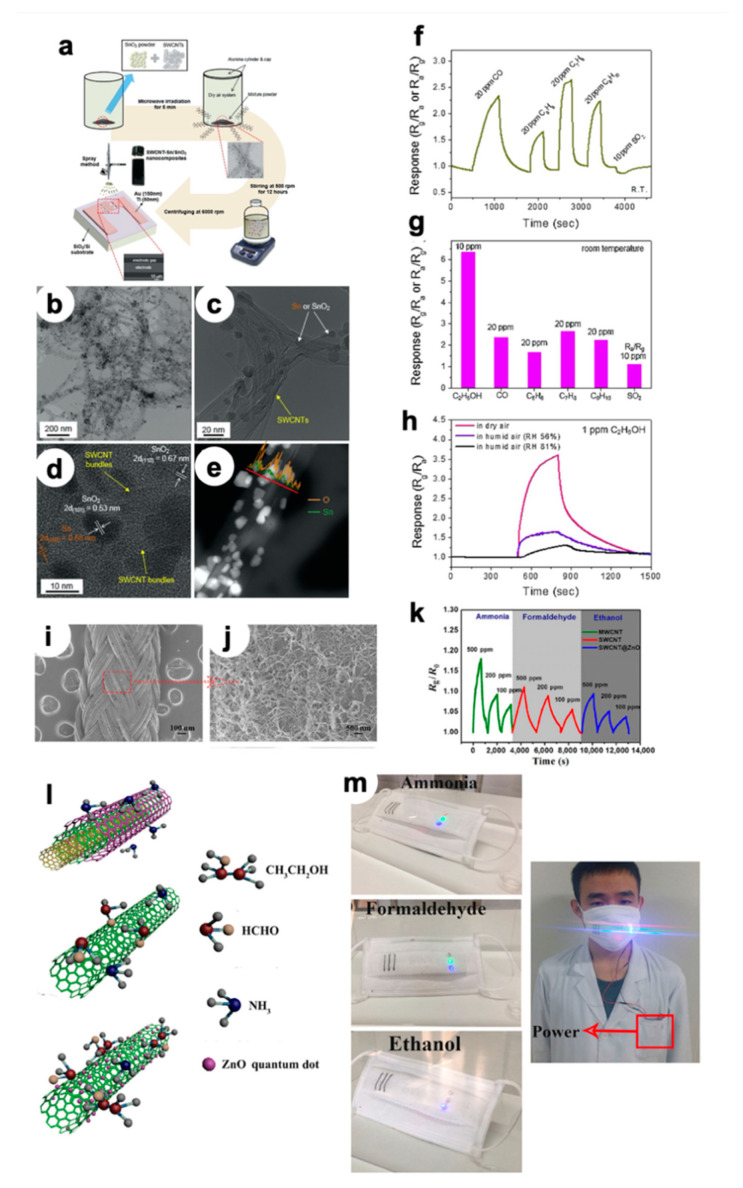
(**a**) Synthetic method used to fabricate holey Single-Wall Carbon NanoTubes (SWCNTs)-Sn/SnO_2_ nanocomposite sensors. The microstructure of the SWCNT-S (with 1:1 SWCNT:SnO_2_) nanocomposite: (**b**) low- and (**c**) high-magnification TEM images, (**d**) lattice-resolved TEM image and (**e**) EDX elemental line profiles of Sn and O around Sn-based nanoparticles. (**f**,**g**) Comparison of gas responses for 20 ppm CO, Benzene Toluene Xylene (BTX), and 10 ppm SO_2_ at RT. (**h**) Comparison of ethanol response of SWCNT-S nanocomposite sensor under dry air and RH 56 and 81% humid air, respectively. Reproduced with permission from [104]. Elsevier, 2019. (**i**,**j**) SEM images of MWCNT-wrapped nylon fibers [109]. (**k**) Response curves of the three fiber-based gas sensors to ammonia, formaldehyde and ethanol, respectively [109]. (**l**) Sensing mechanisms of the three devices selective to the three different gases [109]. (**m**) A multi-functional prototype of face mask with integrated fiber gas sensors. Different colors of LED lights corresponding to the injection of different gases [109].

Instead, as regards Single-Wall CNTs, very recent studies are noteworthy, especially concerning the development of a portable device [2,15,111,112]. In particular, Byoun et al. [108] explored the sensing properties of novel holey SWCNT-Sn/SnO_2_ nanocomposites using microwave (MW) irradiation (Figure 11a) for the detection of ethanol molecules, at room temperature. Specifically, during the synthetic route, defects or open holes are efficiently formed on the sidewalls of SWCNT from collisions between the MWs and atoms in the SWCNTs or SnO_2_ particles. The effective formation of hybrid materials was confirmed by TEM and EDX mapping, as observable in Figure 11b–e. The as-obtained composites were then tested towards several gaseous species, both inorganic and VOCs, evidencing an extremely high response (>6 at 10 ppm) and selectivity for ethanol at room temperature (Figure 11f,g). Such behavior was ascribed to the formation of SWCNT-Sn and SWCNT-SnO_2_ p-n heterojunctions at the interfaces. In addition, these promising materials were also revealed as being useful in humid air (such as a human’s breath), since a perceivable signal to 1 ppm of ethanol was still detectable even under 81% of relative humidity (Figure 11h). Furthermore, Gao et al. [109] succeeded in engineering a lightweight, portable and inexpensive mask for the real-time detection of three gaseous species, i.e., ethanol, formaldehyde and ammonia. Their device comprised the adoption of SWCNT, MWCNT, or ZnO quantum dots-decorated SWCNT (SWCNTs@ZnO) sensing elements. The obtained flexible sensing fibers (Figure 11i,j) could operate at RT, showing good sensitivity, fast response/recovery and, above all, selectivity to the three analytes. 

Specifically, as shown in Figure 11k, MWCNTs could selectively sense ammonia, SWCNTs formaldehyde, whereas SWCNTs@ZnO exhibited boosted sensing behavior towards ethanol molecules. This selectivity tuning was reported to be due to the peculiar surface features of the three different materials, thus leading them to be more affine to a specific target analyte (Figure 11l). Notably, the authors successfully achieved the goal of developing a portable device such as the one displayed in Figure 11m. By integrating the as-synthesized flexible gas sensors into face masks, the fabricated wearable device can selectively sense ethanol, formaldehyde and ammonia by reading the corresponding LEDs with different colors. Hence, such an electronic device can have great potentialities in wearable electronics.

## 6. Conclusions and Future Outlooks

In recent decades, carbon-based materials, such as graphene, its derivatives (GO and RGO), and carbon nanotubes (SWCNTs and MWCNTs), have been adopted as new platforms for the design of innovative metal oxide-based chemoresistors showing boosted sensing features. 

Indeed, thanks to their peculiar properties, such as their unique optical, electrical, mechanical, and thermal properties, they do help the sensitive, selective and lower temperature detection of VOCs. In fact, the current research attention has been focused on the detection of ppm or even ppb concentrations of several volatile organic compounds (like ethanol, acetone, formaldehyde and aromatic compounds), since they are believed to be specific biomarkers of certain human diseases. Hence, their quick detection can represent a useful step for early medical diagnosis.

**Figure 12:**
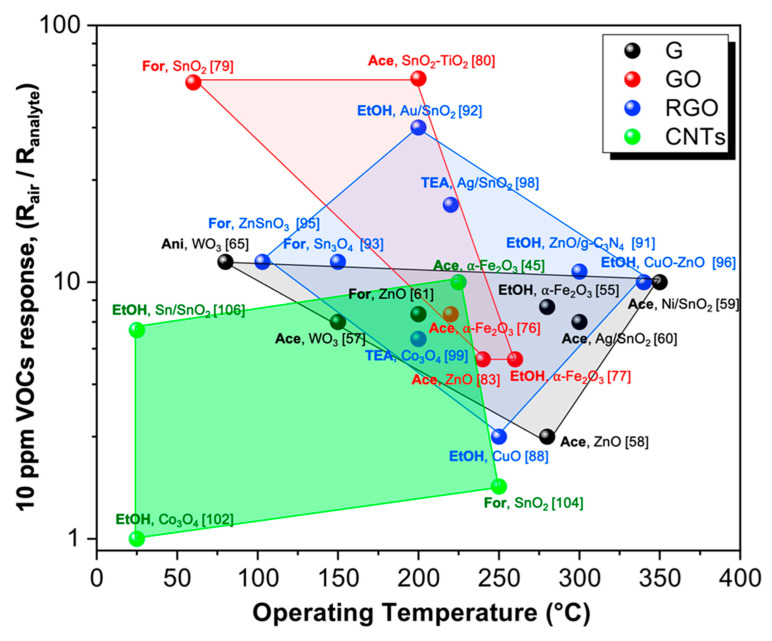
Schematic representation of sensing performances according to the relative optimal operating temperature for the four class of hybrid materials: graphene (G), graphene oxide (GO), reduced graphene oxide (RGO) and carbon nanotubes (CNTs) towards 10 ppm of several VOCs (EtOH = ethanol; Ace = acetone; For = formaldehyde; TEA = triethylamine; Ani = aniline). References have been added in brackets.

Thus, in the present review, we have summarized the main breakthroughs in the engineering of nanocomposites exhibiting excellent sensing behavior in terms of optimal detection limits, selectivity towards specific VOCs, even under humid atmosphere such as a human’s breath. Nevertheless, the majority of the materials recently investigated still have some shortcomings, especially related to the high operating temperatures that hinder their application in portable devices. To overcome this issue, the solutions can include (i) the synthesis of hierarchical, highly porous 3D materials to enhance the active surface area; (ii) the formation of p-n nano-heterojunctions, helping to increase the final signals; (iii) the exploitation of different light sources (such as UV, solar or LED ones) to activate the material enabling the sensing at low T; and (iv) the adoption of carbon nanotubes, which have been reported to show very promising performances at low working temperatures. Figure 12 shows that the above-mentioned carbonaceous matrices in combination with MOS represent possible candidates for detecting VOCs (constant concentration, 10 ppm) at low operating temperatures.

Furthermore, thanks to their flexible structure, CNTs and/or graphene-based materials can be easily implemented in miniaturized hand-held devices. Figure 12 clearly highlights that both 2D materials (especially GO) and CNTs have the best performances in detecting VOCs at low temperatures, and therefore the research attention should be addressed towards these materials. In this respect, we want to stress that there is still some room from improvement, especially concerning the CNTs, since the investigation of their sensing features when coupled to metal oxide nanoparticles is yet less explored; notwithstanding, they seem to have huge potentialities as chemoresistors. In addition, low temperature-operating gas sensors will persist in representing an exciting challenge in the actual era of applications demanding the use of portable systems for point of care diagnosis and so on.

Overall, it is clear from this review that there is a plethora of outbreaks in composite materials to be used in the field of gas sensors; nevertheless, the research will have to continue to go beyond the remaining limits and improve the humans’ health.

## Figures and Tables

**Table 1:** List of some common volatile biomarkers and the relative detection technologies.

Biomarker	Purpose	Levels	Sensor Technology	Ref.
Acetone	Hyperglycemia Diabetes type 1	30 ppb >1800 ppb	Si-doped WO_3_ nanoparticle films	[15,16]
Ammonia	Liver failure	50 ppb	Chemoresistive nano-metal oxides	[17]
		10 ppb	*p-n* oxide semiconductor heterostructures	[18]
2-Butanone	Helicobacter pylori	500 ppb	Chemoresistive graphene-ZnO	[19]
Ethanol/Toluene	Alcohol consumption	<100 ppm	Metal oxides	[20]
H_2_S	Asthma	534 ppt	Chemoresistive	[21]
NO	Lung injury	206 ppt		
C_7_H_14_O, C_10_H_20_O, C_16_H_34_	Ovarian cancer	400 ppb	Chemoresistive flexible metal oxides sensor array	[22]
Trimethylamine	Chronic kidney disease	1.76 ppb	Gas Chromatography-Mass Spectrometry	[23]

**Table 2:** A detailed comparison of graphene (G)–metal oxides (MOS) chemoresistors performances.

Metal oxide@G	Synthetic Route	VOC	Operating T (°C)	LOD (ppm)	Response at LOD (R_air_/R_analyte_)	Response/Recovery Times (s)	Ref.
α-Fe_2_O_3_@(2 wt%)	One-step hydrothermal	Ethanol	280	1	5.0	n.d.	[54]
WO_3_	UV-assisted photoreduction of WO_3_/GO	Alcohols	372	100	~10.0 ^a^	n.d.	[55]
Mesoporous WO_3_	Interface-induced co-assembly process	Acetone	150	2	~3.5^a^	13/65	[56]
ZnO	Hydrothermal	Acetone	280	10	2.8	~2/1	[57]
0.1 wt% Ni-doped SnO_2_	Flame-spray pyrolysis	Acetone	350	2.5	< 5	7/30	[58]
Ag/SnO_2_	Wet-chemical	Acetone	300	0.005	0.007	2/8	[59]
ZnO	Hydrothermal	Formaldehyde	200	2	~10.0 ^a^	10/29	[60]
Fe_3_O_4_	Physical growth	Formaldehyde	150	0.1	< 0.1	n.d.	[61]
SnO_2_	Wet chemical	Benzene	210	0.005	~0.002	n.d.	[62]
Co_3_O_4_	Impregnation followed by calcination	Xylene	26	0.5	~0.18 ^a^	160/235	[63]
WO_3_	Microwave-assisted hydrothermal	Aniline	80	1	~5.0 ^a^	n.d.	[64]

^a^ Extrapolated from the graph.

**Table 3:** A general comparison of graphene oxide (GO)-metal oxides (MOS) chemoresistors performances.

Metal oxide@GO	Synthetic Route	VOC	Operating T (°C)	LOD (ppm)	Response at LOD (R_air_/R_analyte_)	Response/Recovery Times (s)	Ref.
α-Fe_2_O_3_	In situ fluorine directed solution process/heat treatment	Acetone	220	5	< 10.0 ^a^	7/8	[75]
α-Fe_2_O_3_	One-step low temperature hydrothermal	Ethanol	260	5	< 5.0 ^a^	7/6	[76]
SnO_2_	One-step electrospinning	Ethanol	300	50	30.7	n.d.	[77]
Hierarchical SnO_2_	Electrospinning/hydrothermal	Formaldehyde	60	0.25	6.3	n.d.	[78]
SnO_2_	Hydrothermal	Ethanol	25 (UV)	0.1	1.7	310/320	[70]
SnO_2_-TiO_2_	Solvothermal	Acetone	200	0.25	6.3	10/12	[79]
Sn_x_Ti_1-x_O_2_	Hydrothermal	Toluene	350	0.1	< 1.2 ^a^	20–35/30–60	[80]
ZnO	Chemical bath deposition	Acetone	450	10	2.5	~8–12/n.d.	[81]
ZnO	Impregnation	Acetone	240	1	< 2.0	13/7	[82]
ZnO	Hydrothermal	Ethanol	25 (UV)	0.1	~1.1 ^a^	80/90	[83]

^a^ Extrapolated from the graph.

**Table 4:** A detailed comparison of reduced graphene oxide (RGO)-metal oxides (MOS) chemoresistors performances.

Metal oxide@RGO	Synthetic Route	VOC	Operating T (°C)	LOD (ppm)	Response at LOD (R_air_/R_analyte_)	Response/Recovery Times (s)	Ref.
CuO	One-step microwave-assisted	Ethanol	250	5	~0.1^a^	29/n.d.	[86]
WO_3_·0.33H_2_O	Ultrasonic spray nozzle/microwave-assisted hydrothermal	2-propanol	22	1	< 1.5 ^a^	n.d.	[87]
Co_3_O_4_	Hydrothermal	Ethanol	200	20	~3.0 ^a^	n.d.	[88]
ZnO/g-C_3_N_4_	Ultrasonic dispersion/electrostatic self-assembly strategy	Ethanol	300	0.5	< 5.0 ^a^	76/6	[89]
Au/SnO_2_	One-step wet chemical	Ethanol	200	1	~3.0 ^a^	14/10	[90]
Sn_3_O_4_	One-step hydrothermal	Formaldehyde	150	1	~6.0 ^a^	4/125	[91]
Hollow SnO_2_	One-step hydrothermal	Formaldehyde	130	5	~60.0 ^a^	n.d.	[92]
ZnSnO_3_	Self-assembly	Formaldehyde	103	1	~3.0 ^a^	31/n.d.	[93]
CuO-ZnO	One-step hydrothermal	Ethanol	340	10	< 20.0 ^a^	10/14	[94]
WO_3_·0.33H_2_O	One-pot microwave-assisted hydrothermal	Acetone	25	1	~1.0 ^a^	14/n.d.	[95]
Ag/SnO_2_	One-step hydrothermal	Triethylamine	220	0.5	~5.0 ^a^	11/n.d.	[96]
Double-layer Co_3_O_4_	One-step hydrothermal	Triethylamine	200	1	< 4.0 ^a^	30/32	[97]
Pd/ZnO	Sol-gel/electrospinning methods	Benzene	400	1	~23.0 ^a^	100/330	[98]
Au/ZnO					~30.0 ^a^	150/75	

^a^ Extrapolated from the graph.

**Table 5:** An overview on carbon nanotubes (CNTs)-metal oxides (MOS) chemoresistors performances.

Class of CNTs	Metal Oxide	Synthetic Route	VOC	Operating T (°C)	LOD (ppm)	Response at LOD (R_air_/R_gas_)	Response/Recovery Times (s)	Ref.
Multi-Wall	α-Fe_2_O_3_	Hydrolysis/annealing	Acetone	225	0.5	< 2.0 ^a^	2/35	[44]
	Co_3_O_4_	Hydrothermal	Ethanol	25	5	~0.5 ^a^	n.d.	[103]
	CuO	DC magnetron sputtering	Ethanol	407	100	~2.0 ^a^	200/n.d.	[105]
	SnO_2_	Sol-gel	Formaldehyde	250	0.05	~1.0 ^a^	100/90	[106]
	ZnO	Wet-chemical	1-butanol	27	50	~1.0 ^a^	20/30	[107]
Single-Wall	Sn/SnO_2_	Microwave irradiation-assisted	Ethanol	25	1	~3.5 ^a^	250/400	[108]
	ZnO Quantum Dots	Impregnation	Ethanol	25	100	~1.0 ^a^	992/301	[109]

^a^ Extrapolated from the graph.
